# Molecular and neural roles of sodium-glucose cotransporter 2 inhibitors in alleviating neurocognitive impairment in diabetic mice

**DOI:** 10.1007/s00213-023-06341-7

**Published:** 2023-03-04

**Authors:** Iwona Piątkowska-Chmiel, Mariola Herbet, Monika Gawrońska-Grzywacz, Kamil Pawłowski, Marta Ostrowska-Leśko, Jarosław Dudka

**Affiliations:** Department of Toxicology, Faculty of Pharmacy, Medical University of Lublin, Jaczewskiego 8b Street, 20-090, Lublin, Poland

**Keywords:** Sodium–glucose cotransporter inhibitors, Diabetes, Neurotrophins, HIF1α, Gene expression, *Snca*, *Bdnf*, *App*

## Abstract

Diabetes causes a variety of molecular changes in the brain, making it a real risk factor for the development of cognitive dysfunction. Complex pathogenesis and clinical heterogeneity of cognitive impairment makes the efficacy of current drugs limited. Sodium-glucose cotransporter 2 inhibitors** (**SGLT2i) gained our attention as drugs with potential beneficial effects on the CNS. In the present study, these drugs ameliorated the cognitive impairment associated with diabetes. Moreover, we verified whether SGLT2i can mediate the degradation of amyloid precursor protein (APP) and modulation of gene expression (*Bdnf*, *Snca*, *App*) involved in the control of neuronal proliferation and memory. The results of our research proved the participation of SGLT2i in the multifactorial process of neuroprotection. SGLT2i attenuate the neurocognitive impairment through the restoration of neurotrophin levels, modulation of neuroinflammatory signaling, and gene expression of *Snca*, *Bdnf*, and *App* in the brain of diabetic mice. The targeting of the above-mentioned genes is currently seen as one of the most promising and developed therapeutic strategies for diseases associated with cognitive dysfunction. The results of this work could form the basis of a future administration of SGLT2i in diabetics with neurocognitive impairment.

**Supplementary Information:**

The online version contains supplementary material available at 10.1007/s00213-023-06341-7.

## Introduction

Disturbances in cognitive functions and memory appear not only in the course of brain injury, mental illness, and neurological diseases such as Alzheimer’s disease (AD) or Parkinson’s disease (PD) but also in the course of metabolic impairment like diabetes mellitus (DM) (Roberts et al.^2014^; Teixeira et al. 2020; Biessels and Whitmer 2020; Li et al. 2016). Despite highly developed diagnostic tools, the molecular mechanisms underlying the development of cognitive dysfunctions in diabetics are still not fully understood (Sandhir and Gupta 2015). However, it is believed that altering the structure and neurophysiological functions of the brain is a consequence of chronic glucose metabolism impairment, increased inflammation, disrupted blood–brain barrier integrity, intracellular oxidative stress, and mitochondrial dysfunction (Moran et al.^2013^; Moran et al.^2019^; Avogaro et al.^2010^; Etchegoyen et al. 2018; Banks and Rhea 2021; Fournet et al. 2018). Deterioration of cognitive abilities and difficulties in processing and restoring remembered information are also associated with the site and the size of changes in the brain (Cukierman et al. 2005; Biessels et al. 2008; Wei et al. 2016). Studies showed that in diabetic patients, the highest degree of gray matter changes is observed in the medial temporal lobe, anterior cingulate gyrus, and medial frontal lobe, whereas white matter in the frontal and temporal regions (Moretti et al. 2012; Ramanoël et al. 2018; Callisaya et al. 2019). It is worth noting here that a similar distribution of cortical atrophy has also been described in the early stages of Alzheimer’s disease (Pini et al. 2016; Thompson et al. 2003; Infante-Garcia et al. 2016; Manschot et al. 2006; de la Monte and Wands 2008). It is increasingly evident that there is an interplay between the metabolic dysfunction associated with type 2 diabetes mellitus (T2DM) and patient susceptibility to the common development of dementia, specifically Alzheimer’s disease (AD) (Arvanitakis et al. 2004; Biessels et al. 2006; Kopf & Frolich 2009; Zilliox et al. 2016).

Some research showed that T2DM and AD have common pathogenetic mechanisms and hence common clinical features. It was proven that metabolic impairment in the course of diabetes (hyper- and hypoglycemia or insulin resistance) leads to chronic inflammation and pathological protein changes (Götz et al. 2009; Yun et al. 2020). Multiple studies have indicated that patients with T2DM have an increased incidence of alpha-synuclein (SNCA) accumulation, aggregation, and phosphorylation in both the pancreatic β cells and the brain (Martinez-Valbuena et al. 2018; Bassil et al. 2017) which favors by disturbed insulin signaling (Gao et al. 2015) and the fibrilization of amyloid-β and tau, two key proteins in Alzheimer’s disease (Twohig and Nielsen 2019). Research shows that the brain-derived neurotrophic factor (BDNF), neurotrophin-3 (NT3), and neurotrophin-4/5 (NT4/5) are known for their respective roles in neuroprotection and neuronal death (Eggert et al. 2021; Vilar and Mira 2016; Nordvall et al. 2022) and can promote the processing of APP by upregulation of α-secretase, thus protecting the brain from Aβ toxicity (Nigam et al.^2017^; Eggert et al. 2018; Jiao et al. 2016; Mitroshina et al. 2020).

On the other hand, it should not be forgotten that generalized inflammation associated with diabetes modulates neurotrophin functions by reducing their production and limiting their neuroprotective action (Chen et al. 2016; Sastre et al. 2008). Studies also indicate a significant contribution of hypoxia-inducible factor 1α (HIF1α) in modulating the APP (Zhang et al. 2016; Catrina and Zheng 2021; Gunton 2020).

However, the interrelationship between APP, BDNF, NT4, SNCA, or HIF1a signaling and cognitive function in diabetic patients is not fully understood. Taking into account the dynamically increasing incidence of diabetes (Saeedi et al. 2019; Steward 2019; Chiang et al.^2014^) as well as its relationship with patients’ susceptibility to cognitive disorders (Saedi et al. 2016; Srikanth et al. 2020; Pandey and Tamrakar 2019), there is an urgency for new and more effective forms of therapy to reduce the development of cognitive deficits in diabetics. A number of studies have shown that anti-diabetic drugs like metformin, thiazolidinediones, and DPP4 inhibitors are capable of entering the brain after systemic administration and promoting neurogenesis which translates into a clinical improvement of cognitive functions of treated patients (Wium-Andersen et al.^2019^; Zhou et al.^2020^; Patil et al.^2014^; Darsalia et al. 2019; Athauda et al.^2017^). The study by Wium-Andersen et al. (2019) demonstrated that patients with diabetes who used metformin, dipeptidyl peptidase-4 (DPP-4) inhibitors, glucagon-like peptide-1 (GLP-1) agonists, or sodium-glucose cotransporter 2 (SGLT2) inhibitors had lower odds for developing dementia. In addition, a meta-analysis by Zhou et al. (2020) showed that diabetic patients treated with DPP-4 inhibitors had a lower risk of dementia than patients treated with metformin or thiazolidinedione. Mui et al. (2021) reported that diabetic patients taking SGLT2 inhibitors for 5 years had a lower incidence of dementia than those using DPP-4 inhibitors during the same period. Whereas, Athauda et al. (2017) confirmed the beneficial effect of therapy with exenatide-glucagon-like peptide-1 (GLP-1) receptor agonist on motor and cognitive activity in patients with Parkinson’s disease.

Sodium-glucose cotransporter 2 inhibitors (SGLT2i, also called gliflozins or flozins) are one of the newly developed oral anti-hyperglycemic agents used to treat the type 2 diabetes mellitus (Chao and Henry 2010; Heise et al. 2013). Currently, there are four SGLT2 inhibitors marketed in Europe (dapagliflozin (Plosker 2012; Kasichayanula et al. 2014), canagliflozin (Lamos et al. 2013), empagliflozin (Scheen 2014; Scott 2014), and ertugliflozin (Nauck 2014)). This group of drugs is distinguished from other antihyperglycemics in a unique mechanism of action that is based on an increasing glucose excretion in the urine through a reduction in renal reabsorption (Ndefo et al. 2015). This group of drugs is characterized by a high safety profile because their mechanism of action is independent of the function of beta-cells and insulin pathways, which reduces the risk of hypoglycemia in patients (Lin et al. 2014). SGLT2 inhibitors show in addition to beneficial metabolic actions, such as improvements in fasting plasma glucose and lowering blood pressure or body weight; they also have a positive effect on the cardiovascular system and central nervous system (CNS) (Abdelgadir et al. 2018; Al Hamed and Elewa (2020); McMurray et al. 2019; Sa-Nguanmoo et al. 2017). Studies show that sodium-glucose co-transport 2 inhibitors (SGLT2i) have a surprising advantage over other anti-diabetic drugs due to their anti-inflammatory effects within the coronary and cerebral vessels (Liu et al. 2021; Paolisso et al. 2022; Heimke et al.^2022^). Heimke et al. (2022) showed that empagliflozin can reduce LPS-activated inflammation, which is characterized by reduced expression of the pro-inflammatory mediators Nos2, IL6, TNF, and Il1b, by inhibiting the ERK1/2-MAP-kinase pathway in microglia. In addition, SGLT2i prevents cognitive decline and protects synaptic plasticity in the hippocampus (Sa-Nguanmoo et al. 2017). Furthermore, these inhibitors may limit the maturation and secretion of the pro-inflammatory cytokines IL-1β and IL-18 by modulating in microglia the NLRP3 inflammasome, a key pathway in the development of AD (Sim et al. 2021; Kim et al.^2020^).

There is an increasing number of reports suggesting that inhibition of SGLT2 may prevent or even ameliorate impairment of CNS (Enerson and Drewes 2006; Patrone et al. 2014). Even though the initial data indicated that SGLT2 is not expressed in the brain and the therapeutic benefit on cognitive function may be due to the peripheral action of these drugs, recent studies have confirmed SGLT-2 expression in many areas of the brain (hippocampus, cerebellum, etc.), responsible for learning processes, food intake, or glucose homeostasis (Nguyen et al. 2020; Shah et al. 2012; Hummel et al. 2022; Koepsell 2020; Pawlos et al.^2021^; Oerter et al.^2019^). Furthermore, Cinti et al. (2017) showed that ertugliflozin as well as empagliflozin and dapagliflozin studied in this work are characterized by the most selective SGLT2 receptor inhibition potential among all drugs in this group. A case–control study by Wium-Andersen et al. (2019) showed that SGLT2 inhibitors were associated with a lower risk of dementia in treated patients. Also, Siao et al. (2022) noted that patients with T2DM treated with SGLT2 inhibitors had a low rate of diabetic complications as well as an 11% lower risk of dementia development compared with patients not using this group of drugs. Moreover, Mui et al. (2021) reported that diabetic patients taking SGLT2 inhibitors for 5 years had a lower incidence of dementia than those using DPP-4i during the same period. Beneficial mechanisms of SGLT2i also include reduction of pro-inflammatory cytokines, reduction of oxidative stress, reduction of glomerular hyperfiltration, inhibition of advanced glycation end-product signaling (McMurray et al. 2019; Nauck 2014; Yaribeygi et al. 2020; Lee et al. 2019; Brauer et al. 2020). SGLT2i has been shown to improve brain insulin sensitivity in obese rats by reducing inflammation, reducing oxidative stress, with the end result being a strong increase in hippocampal synaptic plasticity (Sa-Nguanmoo et al.^2017^). Amin et al. (2020) supported this, demonstrating that empagliflozin decreased cerebral infarct volume, suppressed neuroinflammation and oxidative stress, as well as reduced neuronal apoptosis in brain tissues of hyperglycemic I/R-injured rats.

In addition to the direct mechanisms of SGLT2i action on CNS, there is increasing evidence pointing to the participation of this group of compounds in the improvement of cognitive functions by the inhibition of acetylcholinesterase activity and increasing the acetylcholine levels (Pawlos et al. 2021; Shaikh et al. 2016; Tahara et al. 2016; Rizvi et al. 2014), or impact on the accumulation of beta-amyloid and neurofibrillary tangles (Shaikh et al.^2016^; Hierro-Bujalance et al. 2020; Wiciński et al.^2020^; Esterline et al. 2020).

Esterline et al. (2020) showed that SGLT inhibition can modulate APP level and production of Aβ, having a key role not only in AD development but also in T2DM. Wiciński et al. (2020) showed that SGLT2 inhibitors reduced the accumulation of Aβ in the cortical region of AD-T2DM mice (APP/PS1xdb/db mice) and brain atrophy. Hierro-Bujalance et al.(2020) showed that empagliflozin (EMP) reduced senile plaque density and the levels of soluble and insoluble amyloid β (Aβ) in the cortex and hippocampus of treated mice in APP/PS1xd/db model (model resembling actual AD pathology). In addition, recent reports also indicate the participation of SGLT2i in the restoration of mTOR signaling, which can be very important in preventing or even reducing the progress of neurodegenerative diseases (Rizzo et al. 2022; Stanciu et al. 2021; Pawlos et al.^2021^).

Moreover, last studies show that treatment with SGLT2i (especially empagliflozin) may have a beneficial effect on cerebral BDNF, a key protein promoting memory and survival of neurons, consequently inhibiting the progression of cognitive disorders (Abdelgadir et al. 2018; Lin et al. 2014, Pawlos et al.^2021^).

Considering the aforementioned reports, we aimed to check whether empagliflozin and dapagliflozin affect the brain cytokine profile (IL1, IL6, and TNFα ) and the levels of proteins such as BDNF, NT3, APP, and HIF1α. We also assessed the expression of genes (*App*, *Bdnf*, *Snca*) involved in the control of neuronal proliferation, plasticity, and memory in the prefrontal cortex and hippocampus of treated mice. In the present study, we intended to examine whether the investigated drugs would be able to ameliorate cognitive impairment by modulating neurochemical parameters and mRNA levels of *Snca*, *Bdnf*, and *App* in the brain. Moreover, we checked whether SGLT2i can mediate the degradation of APP involved in dementia that occurs in T2DM, as well as in AD. This is an intriguing assumption since most of the research on the effects of the SGLT2 inhibitor to date has focused on the effects on the kidneys.

## Materials and methods

### Animals

CD-1 male mice (seven weeks old, 22–25 g) were obtained from a licensed breeder’s animal facility, Experimental Medicine Centre (EMC), Medical University of Lublin, Poland (077—EMC number in Lublin in the Breeders’ Register kept by the Minister of Science and Higher Education, Poland). The animals were housed at 4 individuals per cage with free access to water and food and were kept under constant temperature (20–21 °C ± 1 °C) and humidity (60 ± 10%) and a 12-h light/dark cycle. The number of animals was 8 per group. Animal maintenance and treatments were performed in accordance with binding European standards related to the experimental studies on animal models (Act from January 15, 2015, on the Protection of Animals Used for Scientific or Educational Purposes; Directive 2010/63/EU of the European Parliament and of the council of 22 September 2010 on the protection of animals used for scientific purposes). Procedures were also approved by the Local Ethics Committee at the University of Life Science in Lublin (No. 43/2018, Lublin, Poland). All activities were carried out by qualified staff; the animals were under the constant supervision of the veterinarian; all efforts were made to minimize the anxiety and suffering of mice. The total number of animals was estimated in accordance with the requirements of statistical analyses, the Three Rs (3Rs), and the ARRIVE guidelines (Animal Research: Reporting of In Vivo Experiments).

### Drugs and chemicals

In the experiment, we used streptozotocin (≥ 98% HPLC, Sigma-Aldrich, Munich, Germany) which was freshly prepared in citrate buffer (0.01 M, pH = 4.5), crystalline fructose (Biomus, Lublin, Poland); empagliflozin (Jardiance, Boehringer Ingelheim International GmbH, Germany), and dapagliflozin (Forxiga, AstraZeneca AB, Södertälje, Sweden) dissolved in saline (*aqua pro iniectione*, Baxter, Lublin, Poland). Sodium citrate was supplied by Biomus Company (Lublin, Poland).

### Experimental design – mouse model of diabetes and drug administration

A mouse model of diabetes was used in the experiment. This model was designed and described in detail in our previous article (Piątkowska-Chmiel et al. 2021). In brief, diabetes was induced for 4 weeks by ad libitum administration of 20% aqueous fructose solution to mice, followed by injection of freshly prepared STZ solution (40 mg/kg body weight, *ip*) for 5 consecutive days (1 × daily). Animals with blood glucose ≥ 11 mmol/L were considered diabetic, whereas control mice received only citrate buffer (group I: CTL, *n* = 8).

**Scheme 1:**
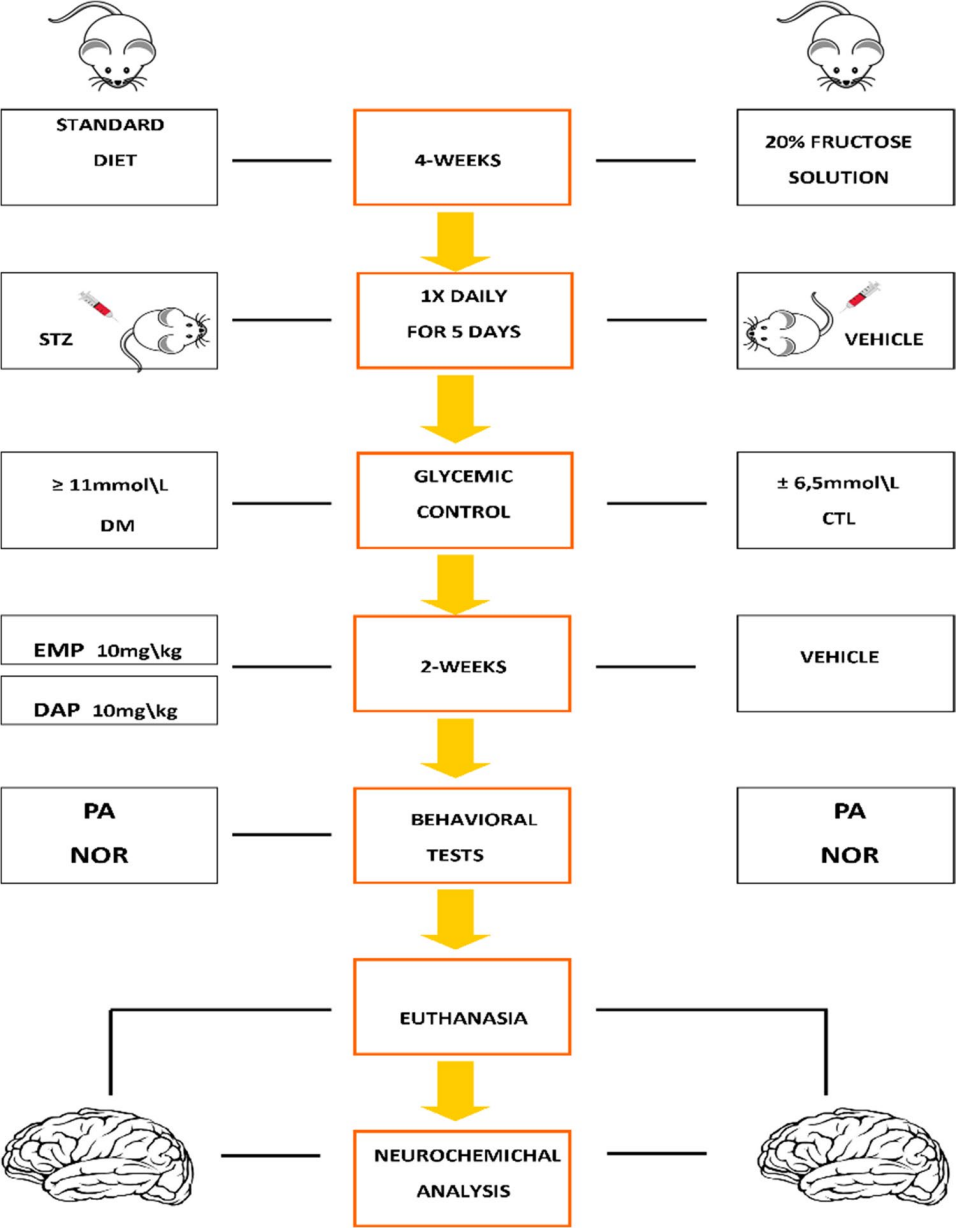
Experimental design of mouse model of diabetes and drug administration

In the next stage, randomly selected animals with induced diabetes were assigned to three groups, each containing 8 mice. They were group II (DM): mice with confirmed diabetes; group III (DM-EMP); and group IV (DM-DAP): mice with confirmed diabetes treated with empagliflozin (EMP) or dapagliflozin (DAP) (10 mg/kg/day, *po*) for 14 days. In this phase of the experiment, animals in the control group (CTL) and those with induced diabetes (DM) received equal volumes of physiologic saline (Scheme 1).

### Behavioral tests assessing memory

#### Passive avoidance test

The short-term and long-term memory of the mice was assessed by a passive avoidance (PA) test. On the first day, one hour after the administration of the last dose of drugs, the mice were placed in a two-compartment step-through passive avoidance apparatus. Both parts were separated by a wall with an 8-cm wide passage. The floor of the dark compartment was composed of 2-mm stainless steel rods spaced 1-cm apart and connected to a constant voltage power source. The animal was placed in the bright area, and next, the researcher waited for it to pass into the darkened area of the apparatus. Then, when the hind legs of the mice entered the dark chamber, the guillotine door was closed, and an electrical foot shock (0.6 mA) was delivered through the grid floor for 2 s. This impulse was a negative training stimulus. After 1 h and after 24 h, the second step of the test was performed. The mice were placed back in the bright compartment to check whether they avoided entering the dark chamber. This time was defined as the latency time, which was recorded for up to 180 s.

#### The novel object recognition test

The novel object recognition (NOR) test is an efficient tool for testing learning and different types of memory in mice through manipulation of the retention interval, i.e., the amount of time elapsed between the training and the testing phase. This test allows the examination of the visual and tactile properties of the explored objects rather than their spatial. The test was carried out based on the method previously described (Piątkowska-Chmiel et al. 2021). A novel object recognition test was conducted in a 40 × 40 × 40 cm white-colored wood box. The test consisted of 4 stages, 5 min each. On the first day, the mice were kept in the empty box to familiarize themselves with the environment (habituation phase). On the second experimental day, mice were allowed to examine two identical objects for 5 min (training phase). Wooden cubes of various shapes (oval, rectangular, or triangular pyramids) were used as objects. After the 1-h break, the testing phase begins. One of the objects was replaced with a new one, and then the time spent by mice in contact with the new object was measured for the different experimental groups to compare the cognitive performance. After 24 h, the animals were placed again in the wood box for 5 min with two objects (familiar and new objects) – second testing phase. As before, the time for animals’ interactions with individual blocks was measured.

The obtained data were calculated and presented as recognition index (%) = (time of exploring the novel object/total exploration time) × 100 (1st, 2nd testing day).

### Neurochemical analysis

One day following the behavioral tests, mice were killed by decapitation. On the day of decapitation, the brain from each animal was removed, and immediately, the prefrontal cortex and hippocampus were isolated for determining the levels of interleukin-1β (IL-1β), interleukin-6 (IL-6), tumor necrosis factor α (TNF-α), brain-derived neurotrophic factor (BDNF) protein, neurotrophin-4 (NT4), hypoxia-inducible factor 1α (HIF1α), β-amyloid precursor protein (APP), as well as gene expression of *App*, *Snca*, and *Bdnf*.

#### Brain samples preparation

Briefly, after isolation, the tissues were rinsed in ice-cold PBS to remove excess blood thoroughly and weighed. Then, the tissues were homogenized in fresh lysis buffer (w:v = 1:50) on ice. The resulting suspension was sonicated with an ultrasonic cell disrupter until the clear solution. Next, homogenates were centrifuged at 10,000 g for 5 min at 4 °C to obtain supernatants, which were stored at − 20 °C until use. Total protein concentrations for all homogenates were assayed using the Bradford method (Bradford 1976).

#### Measurement of cytokines’ and proteins’ (BDNF, NT4, APP, HIF1α) levels

The concentrations of cytokines in supernatants were assessed by enzyme-linked immunosorbent assay (ELISA kits for mice: interleukin IL-1*β*, IL-6, TNF*α*, BDNF, NT4, APP, and HIF1*α*; Cloud-Clone Corp., Houston, TX, USA). Each parameter was determined individually in all samples according to the manufacturer’s protocols. The concentrations of cytokines and proteins such as BDNF, NT4, APP, and HIF1*α* were determined by comparing the optical density of the samples to the standard curve. Cytokines’ concentrations and the levels of BDNF, NT4, APP, and HIF1*α* in the prefrontal cortex were expressed in picograms per ml/mg protein.

### RNA extraction and real-time PCR

The analysis of mRNA expression was determined as previously described (Piątkowska-Chmiel et al. 2021). Briefly, the RNA was isolated from both the prefrontal cerebral cortex and hippocampus of mice using TRIzol Reagent (Invitrogen, Carlsbad, CA, USA). The concentration and purity of RNA were measured spectrophotometrically with a NanoDrop MaestroNano spectrophotometer (Maestrogen, Hsinchu, Taiwan). RNA with A260/280 ratio ranging between 1.8 and 2.0 was used for further investigations. cDNA was synthesized with a High Capacity cDNA Reverse Transcription Kit (Applied Biosystems, Foster City, California, USA) according to the manufacturer’s instructions. The mRNA expression levels of *App*, *Bdnf*, and *Snca* genes involved in the inflammatory response were measured by a real-time PCR reaction and ^ΔΔ^Ct method, using *Hprt* and *Tbp* as endogenous controls (for details, see Table 1). The reaction was carried out in triplicates using the 7500 Fast Real-Time PCR System (Applied Biosystems, Foster City, California, USA) and Fast Probe qPCR Master Mix (2 ×), plus ROX Solution (EURx, Poland) according to the manufacturer’s protocol. The data quality screen based on amplification, Tm, and Ct values was performed to remove any outlier data before ^ΔΔ^Ct calculations and to determine fold change in mRNA levels. The data were presented as RQ values.

**Table 1:** Data on the primers used: gene symbols, assay IDs, gene names, GenBank references sequence accession numbers, and amplicon lengths (bp)

Symbol of the gene	Assay ID	Name of the gene	Ref seq	The length of amplicon (bp)
*App*	AB ID: Mm01344172_m1	Amyloid beta (A4) precursor protein	NM_001198823.1 NM_001198824.1 NM_001198825.1 NM_001198826.1 NM_007471.3	111
*Bdnf*	AB ID: Mm01334047_m1	Brain-derived neurotrophic factor	NM_007540.4	105
*Hprt*	AB ID: Mm00446968_m1	Hypoxanthine guanine phosphoribosyl transferase	NM_013556.2	65
*Snca*	AB ID: Mm01188700_m1	Synuclein, alpha	NM_001042451.2 NM_009221.2	67
*Tbp*	AB ID: Mm00446974_m1	TATA box binding protein	NM_013684.3	105

*AB ID*, applied biosystems TaqMan GENE EXPRESSION ASSAY ID

### Statistical analysis

All statistical parameters were calculated using GraphPad Prism version 8.0.1 (GraphPad Prism, San Diego, CA, USA). All of the data were analyzed by a one-way ANOVA, followed by Tukey’s post hoc test for analysis of significance. Differences with a *p*-value less than 0.05 were considered statistically significant. No outliers were removed from the dataset. Data are expressed as mean ± standard error of the mean (SEM).

## Results

### Protective effects of empagliflozin and dapagliflozin on diabetes-induced cognitive deficits

**Fig. 1:**
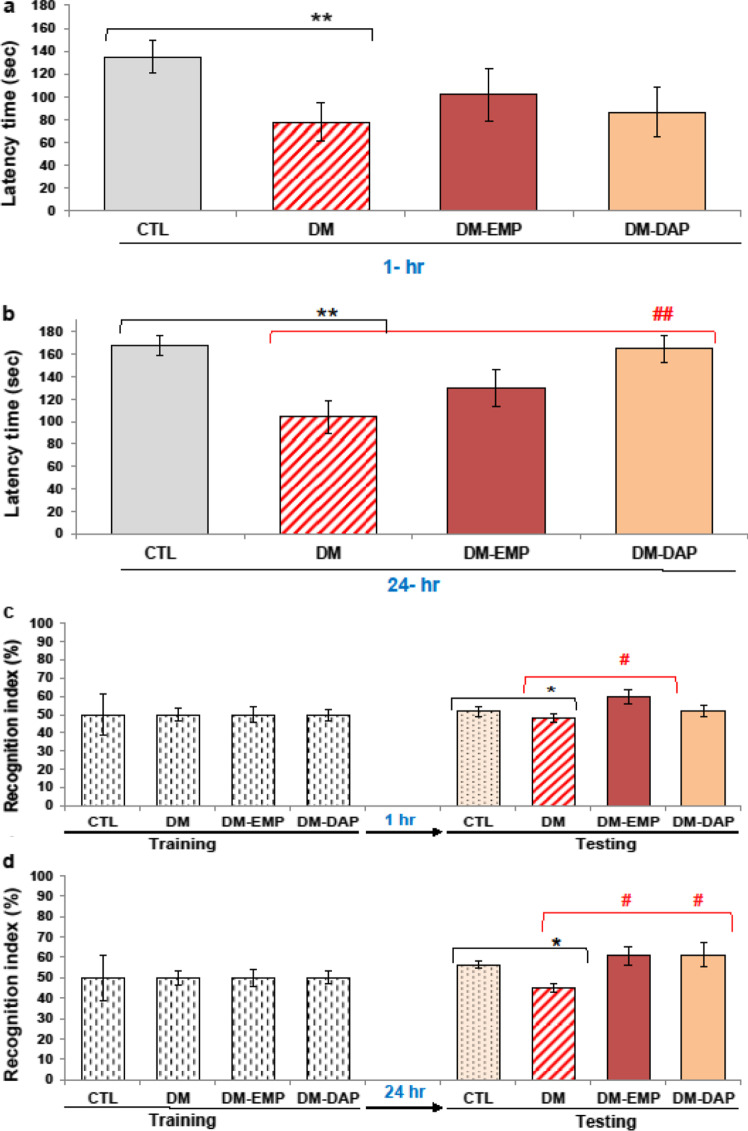
Effect of empagliflozin and dapagliflozin on diabetes-induced neurobehavioral deficits. Cognitive function was determined using a passive avoidance (PA) task (**a**, **b**). Novel object recognition (NOR) test was performed to evaluate the memory function (**c**, **d**). CTL: control group; DM: mice with confirmed diabetes; DM-EMP: mice with confirmed diabetes treated with empagliflozin (EMP) (10 mg/kg/day, *per os*) for 14 days; DM-DAP: mice with confirmed diabetes treated with dapagliflozin (DAP) 10 mg/kg/day, *per os*) for 14 days. *p < 0.05, **p < 0.01, as compared with the control group (CTL). ^#^*p* < 0.05, ^##^*p* < 0.01 as compared with the untreated diabetic group (DM) (one-way ANOVA, followed by Tukey’s post hoc test). Bars represent means ± SEM, *n* = 8/group

Analysis of data showed that the investigated drugs had a moderate impact on short-term memory deficits at levels to be detected by the selected behavioral tests (Fig. 1a–d). As can be seen in Fig. 1a, there was not any significant difference in latency time (the time to enter the dark compartment of the apparatus) determined in the passive avoidance test in the groups of sodium-glucose cotransporter 2 inhibitors-treated mice when compared with diabetic mice (*p* > 0.05). The slightly longer latency time was observed in empagliflozin-treated animals (DM-EMP) compared to diabetic mice (DM) in the PA test performed after an hour, but it was not statistically significant (Fig. 1a, p > 0.05). Whereas the NOR test revealed that empagliflozin (EMP) led to a significant improvement in the short-term memory of treated animals (Fig. 1c, p < 0.05, *F*_(4.34)_ = 2.291) as well as long-term memory (Fig. 1d, p < 0.05, *F*_(4.34)_ = 3.638). The recognition index (RI) in the DM-EMP group was 59.8% in the case of tests performed after 1 h and 60.7% after 24 h which indicated a significant preference of animals for the novel object as opposed to untreated mice (Fig. 1c,d). For comparison, the recognition index (%) in diabetic mice was significantly below 48.2–44.5%, suggesting that the group of animals had recognition memory deficits.

In turn, treatment with dapagliflozin (DAP) significantly retrieved the long-term memory functions in treated mice which was evidenced by an increase in latency time during the PA test performed after 24 h (Fig. 1b; *p* < 0.01, *F*_(4.34)_ = 5.001; a 1.5-fold increase of the latency) and by a level of recognition index (RI) in NOR test (Fig. 1d, p < 0.05, *F*_(4.34)_ = 3.638) in comparison to untreated diabetic mice. The value of the recognition index (%) in treated mice was significantly above 61%, which indicates that they were able to distinguish between presented objects, as opposed to untreated mice. For comparison, RI in diabetic mice was significantly below 50% (Fig. 1d, p < 0.05, *F*_(4.34)_ = 3.638), suggesting that this group of animals had recognition memory deficits.

It should be highlighted that there was no significant difference in recognition index in the training phase between any of the groups (mean ± SEM).

### Effect of empagliflozin and dapagliflozin on the levels of inflammatory cytokines in the brain of diabetic mice

**Fig. 2:**
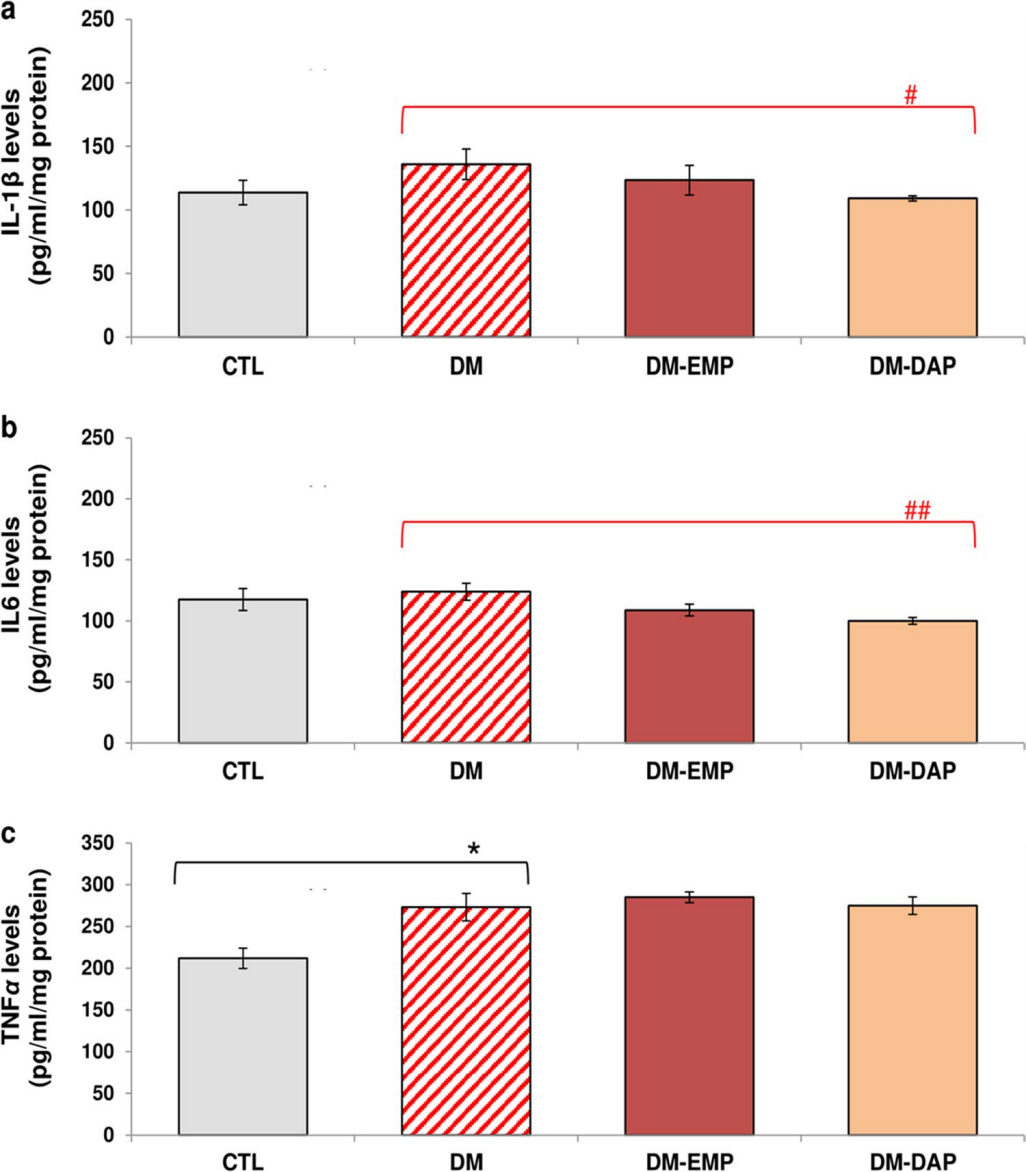
Effect of empagliflozin and dapagliflozin on the brain cytokines profile of diabetic mice. The levels of cytokines, IL-1*β* (**a**), IL6 (**b**), and TNF*α* (**c**), in prefrontal cortex homogenates were assessed by using commercially available mouse enzyme-linked immunosorbent assays (ELISA). CTL: control group; DM: mice with confirmed diabetes; DM-EMP: mice with confirmed diabetes treated with empagliflozin (EMP) (10 mg/kg/day, *per os*) for 14 days; DM-DAP: mice with confirmed diabetes treated with dapagliflozin (DAP) 10 mg/kg/day, *per os*) for 14 days. Comparisons between groups were made by a one-way ANOVA, followed by Tukey’s post hoc test. Bars in the figures present the means ± SEM, *n* = 8/group. ^#^*p* < 0.05, ^##^*p* < 0.01 as compared with the untreated diabetic group (DM)

As shown in Fig. 2a–c, in the diabetic group (DM), the statistically significant changes in the levels of pro-inflammatory cytokines were not recorded with the exception of TNF*α* compared to healthy animals (CTL).

Among the tested drugs, the 14-day treatment with dapagliflozin (DAP) affected the concentrations of pro-inflammatory cytokines in the prefrontal cortex of diabetic mice. As shown in Fig. 2a,b, DAP significantly decreased (by about 20%) the level of IL-1*β* and IL6 (*p* < 0.01, *F*_(2.94)_ = 1.878; *p* < 0.001, *F*_(2.94)_ = 2.865, respectively) in comparison to diabetic mice group (DM). In addition, 14-day therapy with EMP and DAP failed to reverse the increased TNFα level in the prefrontal cortex of diabetic mice (Fig. 2c; *p* > 0.05).

### Attenuation of cognitive deficits through the restoration of neurotrophins’ levels

**Fig. 3:**
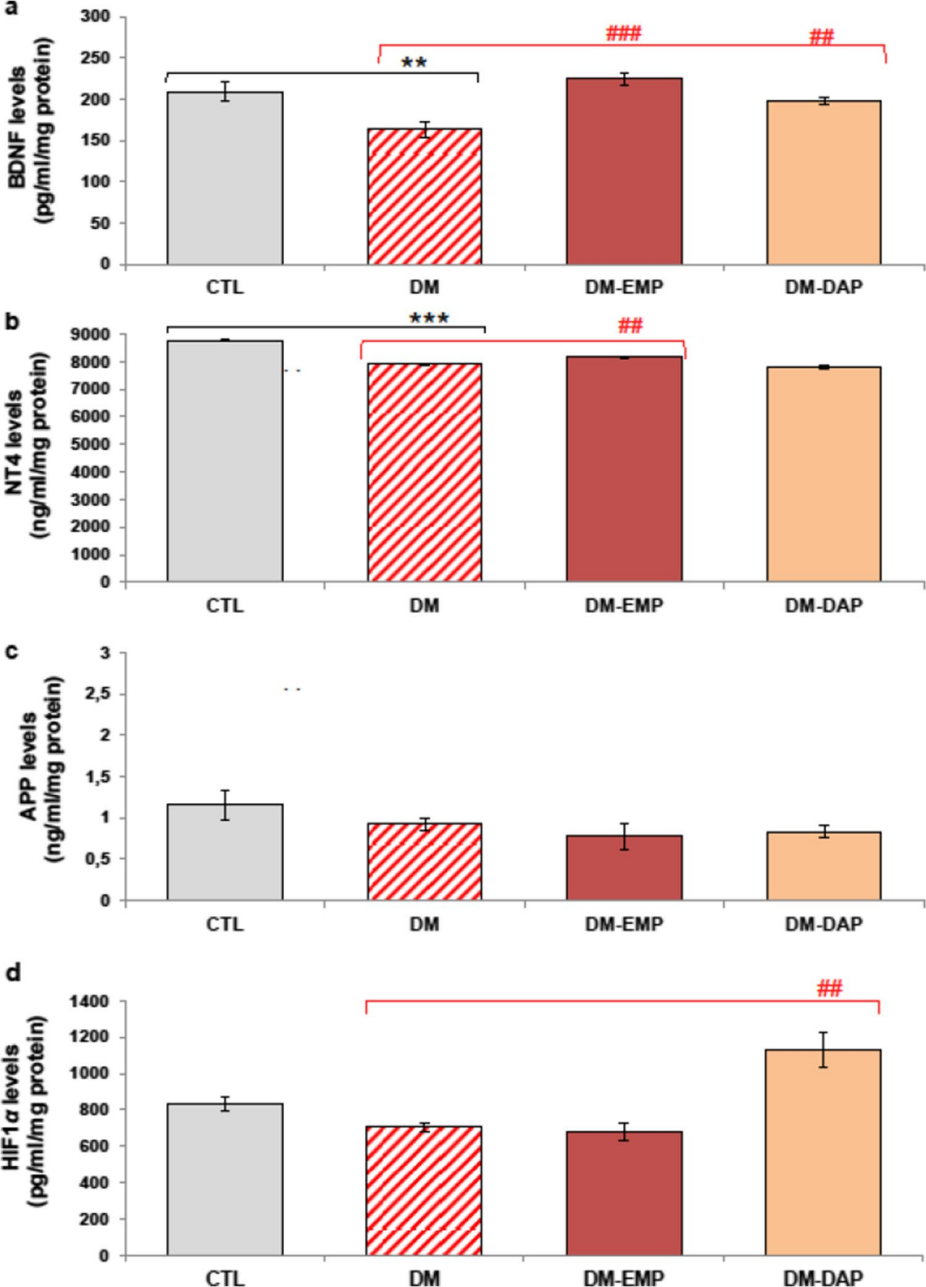
Effect of empagliflozin and dapagliflozin on the levels of neurotrophins (BDNF, NT4), APP, and HIF1*α *in brain of diabetic mice. BDNF (**a**), NT4 (**b**), APP (**c**), and HIF1*α* (**d**) protein levels in prefrontal cortex homogenates were measured using commercially available mouse enzyme-linked immunosorbent assays (ELISA). DM-EMP: mice with confirmed diabetes treated with empagliflozin (EMP) (10 mg/kg/day, *per os*) for 14 days; DM-DAP: mice with confirmed diabetes treated with dapagliflozin (DAP) 10 mg/kg/day, *per os*) for 14 days. Comparisons between groups were made by a one-way ANOVA, followed by Tukey’s post hoc test. **p < 0.01, as compared with the control group (CTL). ^##^*p* < 0.01, ^###^*p* < 0.001 as compared with the untreated diabetic group (DM)

 As can be seen in Fig. 3a, the mice receiving empagliflozin and dapagliflozin at a dose of 10 mg/day had significantly increased levels of brain BDNF compared with the untreated diabetic mice (*p* < 0.001, *F*_(2.94)_ = 11.437; *p* < 0.01, *F*_(2.94)_ = 11.437, respectively). In addition, a one-way ANOVA with Tukey’s post hoc test, as seen in Fig. 3b, revealed that the fourteen-day therapy with empagliflozin led to an increase in the level of the NT4 protein in the prefrontal cortex of treated mice compared with diabetic animals (*p* < 0.05, *F*_(2.94)_ = 4.382). As can be seen in Fig. 3c, the APP protein level for the EMP-or DAP-treated group was not statistically significant when compared with the untreated diabetic group (*p* > 0.05).

The results of this study also indicate a significant impact of dapagliflozin on the level of HIF1*α* in the brain of treated mice compared with the untreated animals (Fig. 3d, p < 0.01, *F*_(2.94)_ = 5.405). As can be seen in Fig. 3c,d, fourteen-day therapy with empagliflozin did not show a significant impact on either the APP protein level or the level of HIF1*α* in the brain of treated mice (*p* > 0.05) when compared with diabetic mice.

### Empagliflozin and dapagliflozin significantly improved the mRNA expression levels of neurotrophic factor and neuronal proteins

**Fig. 4:**
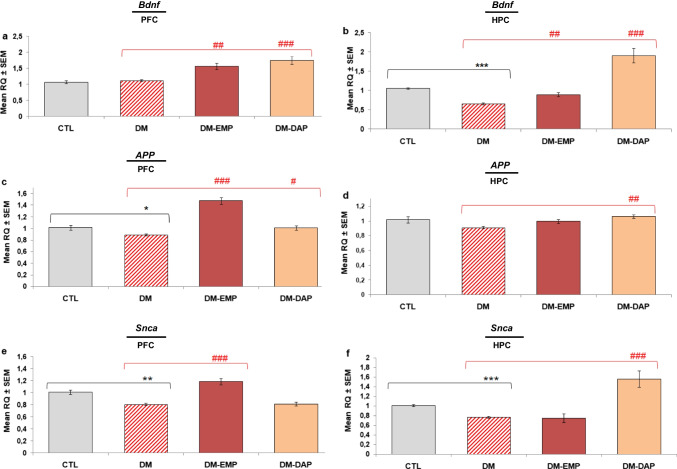
The effect of empagliflozin and dapagliflozin on the mRNA expression of *Bdnf*, *App*, and *Snca* in the brain of diabetic mice. *Bdnf* (**a**, **b**), *App* (**c**, **d**), and *Snca* (**e**, **f**) in the prefrontal cortex (PFC) and hippocampus (HPC) were analyzed by quantitative real-time PCR (qRT-PCR). Data are expressed as the means ± SEM, *n* = 8/group. CTL: control group; DM: mice with confirmed diabetes; DM-EMP: mice with confirmed diabetes treated with empagliflozin (EMP) (10 mg/kg/day, *per os*) for 14 days; DM-DAP: mice with confirmed diabetes treated with dapagliflozin (DAP) 10 mg/kg/day, *per os*) for 14 days. Comparisons between groups were made by a one-way ANOVA, followed by Tukey’s post hoc test. *p < 0.05, **p < 0.01, ***p < 0.001 as compared with the control group (CTL). ^#^*p* < 0.05, ^##^*p* < 0.01, ^###^*p* < 0.001 as compared with the untreated diabetic group (DM)

To examine the effect of empagliflozin and dapagliflozin on the gene expression of *App*, *Bdnf*, and *Snca*, mRNA levels were analyzed by quantitative real-time PCR (qRT-PCR) in the samples of the prefrontal cortex and hippocampus of diabetic mice. As shown in Fig. 4a,b, the administration of empagliflozin and dapagliflozin caused the increased expression of *Bdnf* in the prefrontal cortex (*p* < 0.01, *F*_(2.94)_ = 17.323; *p* < 0.001, *F*_(2.94)_ = 17.323, respectively) and hippocampus (*p* < 0.01, *F*_(2.94)_ = 30.509; *p* < 0.001, *F*_(2.94)_ = 30.509, respectively). The expression levels of the studied genes were generally lower in the prefrontal cortex and hippocampus of diabetic mice (Fig. 4a–f). The expression of *App* mRNA, analyzed in the prefrontal cortex by qRT-PCR, was significantly increased in the empagliflozin- or dapagliflozin-treated group in comparison to the diabetic group (Fig. 4c; *p* < 0.001, *F*_(2.29)_ = 42.579; *p* < 0.05, *F*_(2.29)_ = 42.579, respectively). Whereas *App* gene expression level in the hippocampus significantly increased only in the animal group treated with dapagliflozin (Fig. 4d, p < 0.01, *F*_(2.94)_ = 4.809) in comparison to diabetic mice. Moreover, we observed a significant increase in *Snca* gene expression level in hippocampal structure in mice treated with dapagliflozin compared with the untreated group (Fig. 4f; *p* < 0.001, *F*_(2.94)_ = 18.633). Whereas empagliflozin had no significant effect on *Snca* gene expression in the above-mentioned mouse brain structure (*p* > 0.05), but significantly up-regulated it in the prefrontal cortex. As can be seen in Fig. 4e, the mRNA level of *Snca* was significantly higher in animals treated with empagliflozin than in the group of diabetes-induced mice (*p* < 0.001, *F*_(2.94)_ = 24.643). In turn, dapagliflozin had no significant effect on the *Snca* gene expression in the prefrontal cortex of treated mice (*p* > 0.05).

## Discussion

Our previous research (Piatkowska-Chmiel et al. 2021; Piątkowska-Chmiel et al. 2022a, 2022b) as well as the results of other scientists (Exalto et al. 2012; Chatterje et al. 2016; Li et al. 2015; Schuh et al. 2011; Jayaraj et al. 2020) confirm the relationship between diabetes and increased risk of cognitive dysfunction, dementia, and even Alzheimer’s disease. The presented study also proves this dependence. Diabetic mice showed significant memory and learning impairment in behavioral tests. Additionally, they were characterized by decreased levels of neurotrophins, the down-regulation of *Snca*, *App*, and *Bdnf* gene expression, and the increased concentration of pro-inflammatory TNF*α* in the brain.

However, after 2 weeks of administration of empagliflozin or dapagliflozin, learning, and memory dysfunctions have been improved in mice with diabetes. Our results are consistent with the observations of other scientists. Studies have proven that the SGLT2 protein is present in some regions of the brain including the cerebellum and hippocampus with complex functions in the CNS (Yu et al.^2010^, ^2013^). Both empagliflozin and dapagliflozin revealed an overall improvement in learning and memory in animal models of dementia and T2DM (Sa-Nguanmoo et al. 2017; Lin et al.^2014^; Hierro-Bujalance et al. 2020; Mousa et al.^2023^). The studies on neuroimaging and neuropathology showed a reduced risk of developing dementia in EMP-treated patients which may be due to the protective effect of the drug on cerebrovascular endothelial cells (Hayden et al. 2019; Lin et al. 2014; Bello-Chavolla et al.^2019^). Animals treated with SGLT2i showed histological improvement in neurovascular restructuring which is associated with cognitive decline (Hayden et al. 2019). The data demonstrated that combination therapy of dapagliflozin and liraglutide improved the cognitive function in dietary-induced diabetic mice by the increased number of neurons in the dentate gyrus and synaptophysin (Millar et al.^2017^). Furthermore, diabetic patients treated with SGLT2 inhibitors had a lower rate of diabetic complications as well as a lower risk of development of dementia compared with patients using other groups of anti-diabetic drugs (Siao et al. 2022; Mui et al. 2021). SGLT2i seem to be more effective in improving hippocampal synaptic plasticity than other drug classes such as dipeptidyl peptidase-4 (DPP4) inhibitors, through reduced oxidative stress, better insulin signaling, and increased synaptic activity in the hippocampus (Sa-Nguanmoo et al. 2017; Hierro-Bujalance et al. 2020). Moreover, Sa-Nguanmoo et al. (2017) showed that dapagliflozin had greater efficacy in improving insulin signaling and hippocampal synaptic plasticity than vildagliptin. Significant improvements in learning and memory processes were noted in the treated animals. Arab et al. (2021) noted that dapagliflozin counteracted neuronal apoptosis and up-regulated glial cell-derived neurotrophic factor (GDNF) by lowering lipid peroxidation.

The role of neurotrophins in normal neural development or response to traumatic brain injuries is widely described in the literature (Srikanth et al. 2020; Jiao et al. 2016). Therapies that improve neurotrophic factors’ levels could efficiently neutralize the effects of oxidative damage, microglia activation, and apoptosis while promoting neuron regeneration and synaptogenesis. All these actions translate into the improvement of learning and memory formation (Vilar and Mira 2016; Nordvall et al. 2022; Rex et al. 2007). Mice with diabetes given empagliflozin or dapagliflozin showed a significant increase in BDNF and NT4 protein levels in the prefrontal cortex. Although the mechanisms of the pro-cognitive action of SGLT2i have not been fully explained, based on the collected data, it can be hypothesized that restoration of the brain’s neurotrophin levels and gene expression involved in neural plasticity attenuated behavioral deficits observed in diabetic mice in our research. The results of our observations are consistent with those of other scientists. Lin et al. (2014) observed attenuation of cerebral oxidative stress and an increase in BDNF in EMP-treatment diabetic mice; these effects were also accompanied by an improvement in cognitive function. Furthermore, at the moment, we know that the observed improvement in cognitive functions in EMP- or DAP-treatment mice is not due to the modulation of the level of molecules such as IL-1beta or IL-6.

Multiple studies have shown that SGLT2i (particularly empagliflozin and luseogliflosin) can improve cognitive functions by reversing cerebrovascular dysfunction in animals and diabetic subjects (Wang and Fan 2019; Wang et al. 2022). It cannot be ruled out that the improvement in the cognitive function we observed in the treated mice is the result of a more complex mechanism of action of these antidiabetic drugs. Some research shows that the neuroprotective effect of SGLT-2is may be related to the increased native GLP-1 concentration which is involved in the control of synaptic plasticity and the course of signaling pathways related to learning and memory (Millar et al. 2017; Yildirim Simsir et al. 2018).

Moreover, in our study, we noted up-regulated gene expression involved in neural plasticity in animals that were EMP- or DAP-treated. In animals treated with EMP, changes in the expression of *Bdnf*, *App*, and *Snca* genes were observed mainly in the prefrontal cortex. Whereas in the group of animals treated with DAP, changes in gene expression were noted in both examined brain structures. Even though we cannot pinpoint a specific mechanism involved in the modulation of expression of the above-mentioned genes, we can hypothesize that the differences in pharmacological effects between the tested drugs may be associated with differences in their chemical structure, duration of action, and distribution level (Tahara et al. 2016). On the other hand, we cannot ignore information that SGLT2i can be dual inhibitors of SGLT2 and acetylcholinesterase (AChE) (Shaikh et al. 2016; Arafa et al. 2017). They may be as effective in inhibiting AChE as galanthamine (Panchal et al. 2018). Mice receiving SGLT2i showed significantly lower levels of AChE, which obviously correlated with greater acetylcholine availability and improved cognitive function (Arafa et al. 2017).

Clinical and preclinical studies suggest that diabetes and Alzheimer’s disease may share many biochemical features and signaling pathways. It has been shown that in the course of these diseases may appear some pathologic forms of proteins both on the periphery and in the brain (Götz et al. 2009; Yun et al. 2020; Martinez-Valbuena et al. 2018; Bassil et al. 2017; de Nazareth 2017; Shinohara and Sato 2017; Matsuzaki et al. 2010; Oskarsson et al. 2015). This phenomenon is favored by impaired glucose metabolism and insulin signaling, resistance to insulin-like growth factor (IGF-1), oxidative stress, neuroinflammation, as well as cerebrovascular dysfunction (Gao et al. 2015; Luchsinger et al. 2001; Ou et al.^2018^). Our data show that the expression of amyloid precursor protein may be reduced in specific brain areas that are known to degenerate in AD. Since APP plays an important role in synapse formation and synaptic plasticity, it is easy to predict that the decrease in expression in diabetic mice has a significant impact on cognitive function. Furthermore, the altered expression of the *App* gene in diabetic animals may affect glucose and insulin homeostasis and thus the functions of the CNS (Unno et al.^2020^). Recent studies have suggested that antidiabetic drugs may reduce amyloid pathology (Infante-Garcia et al. 2016; Holscher 2014; Michailidis et al. 2022; Ou et al. 2018). Interestingly, the studied group of anti-diabetic drugs increased the level of APP mRNA expression without significant influence on the level of protein in the brain of treated mice. The potential reason for the lack of a correlation between mRNA expression and protein level may be delays in their synthesis. Protein synthesis takes time, so transcript changes affect protein levels but with a time lag, which is also seen in our research (Maier et al.^2009^; Liu et al.^2016^). We already know that the control of amyloid precursor protein processing is important not only in the course of Alzheimer’s disease (Eggert et al. 2018) but also in diabetes mellitus (Eggert et al. 2021; Catrina and Zheng 2021). Hierro-Bujalance et al. (2020) reported that empagliflozin reduced senile plaque density, with an overall reduction in soluble and insoluble amyloid β levels in the cortex and hippocampus of mice APP/PS1xdb/db. Therefore, APP processing control by sodium-glucose cotransporter 2 inhibitors may play a pivotal role in disease-modifying therapy for Alzheimer’s disease but also diabetes mellitus. We suppose that, as observed by us, increasing brain expression of *App* in SGLTi-treated mice affected the reduction of the APP turnover rate. Thus, our study partly revealed molecular mechanisms underlying the potential of these drugs for reducing the incidence of dementia and/or AD in people with T2DM.

Research shows that alpha-synuclein (*Snca*) can also be involved in the pathophysiology of neurodegenerative disorders (Twohig and Nielsen 2019; Dawson and Dawson 2003; Yu et al. 2007) as well as pathological processes in the brain of diabetics. Studies showed that observed in the course of diabetes, the decreased tissue glucose uptake, increased insulin resistance, and accelerated neuronal dysfunction are associated with the strong decline of *Snca*, *Arc*, and *Npas4* genes expression levels (Micheli et al. 2021; Piatkowska-Chmiel et al. 2021; Hong et al. 2020). The increase of *Snca* expression slowed down the development of cognitive decline in diabetic mice, indicating that the brain *Snca* level could be an important marker of cognitive impairment progression. The studies confirm that the reduced proliferation of neurogenic niches cells, observed physiologically also during aging, is accompanied by low SNCA expression (Micheli et al. 2021). In our research, a decrease of *Snca* mRNA levels in the hippocampus and prefrontal cortex of diabetic mice was noted in comparison to healthy mice, which was consistent with the results of behavioral tests. Interestingly, after 14 days of treatment with sodium-glucose cotransporter 2 inhibitors, a significant increase in *Snca* gene expression level in brain structures of treated mice was observed when compared with the untreated group. Moreover, the mice characterized by high *Snca* mRNA expression levels demonstrated improved cognitive function in the PA test and NOR test. Arab et al. (2021) showed that dapagliflozin attenuated ROS production, enhanced glial cell lineage-derived neurotrophic factor, preserved dopaminergic neurons, and reduced alpha-synuclein accumulation.

Accumulating evidence suggests that the high glucose levels in diabetes may disrupt the regulation of HIF-1 signaling in tissues, possibly causing complications in the functioning of the nervous system (Catrina et al. 2004), retina (Catrina and Zheng 2021), heart (Marfella et al. 2004), blood vessels (Katavetin et al. 2006), as well as kidney (Gunton 2020). More and more studies show that pharmacological induction of HIF-1 is beneficial for the prevention of the progression of complications in the course of metabolic diseases such as diabetes (Sugahara et al. 2020; Dodd et al. 2018; Rojas et al.^2018^; Zhu et al. 2019; Zeinivand et al. 2020). The results of our study indicate that dapagliflozin is able to modulate hypoxia-inducible factor 1α (HIF1*α*) level in the brain of treated mice when compared with the untreated animals. Research proves that the enhanced HIF1*α* expression facilitates glucose metabolism, counters oxidative stress, and improves cerebral blood flow which ultimately contributes to neuronal cell protection and improved cognitive function (Soucek et al. 2003; Silva et al. 2013). Furthermore, HIF1*α* can also downregulate the receptors for inflammatory cytokines in the hippocampus, reducing neuroinflammation (Xing and Lu 2016). Fine et al. (2012) and Sorond et al. (2015) proved that the upregulation of HIF1*α* and its target genes can improve memory by the increase of cerebral blood flow.

In conclusion, our data suggest that diabetes may negatively affect neurons by modulating the neurotrophin levels as well as by the down-regulation of *Snca*, *Bdnf*, and *App* genes expression involved in the control of neuronal functions. Thus, the studies reported here proved a crucial and previously undocumented feature of diabetes pathophysiology; 14-day treatment with empagliflozin or dapagliflozin positively influenced neurochemical parameters including neurotrophin levels and neuronal gene expression (Supplementary Table 2). The results of this study are the first step in the evaluation of the role of SGLT2i in the multifactorial process of neuroprotection. Despite these interesting new discoveries, more research is needed to fully understand the molecular mechanisms underlying the beneficial impact of cognitive functions of this group of drugs in the human population. One limitation of this study is that we have no direct evidence that the modulation of the above-mentioned molecules’ levels by EMP and DAP contributes to the amelioration of cognition. In addition, it would be appropriate to investigate whether the achieved concentrations of drugs in the brain are sufficient to suppress SGLT2. We cannot rule out that the observed improvement of cognitive functions in treated animals is the result of synergistic and complex mechanisms of SGLT2i action. They can act by inhibiting SGLT2, inhibiting the acetylcholinesterase enzyme, reducing the level of oxidative stress and inflammation, or limiting the remodeling of brain vessels. In our opinion, SGLT2i are promising candidates in the treatment of neurocognitive disorders.

## Supplementary Information

Below is the link to the electronic supplementary material.

Supplementary file1 (DOCX 21 KB)

## Data Availability

Not applicable.
