# Supplementary material for: Molecular and neural roles of sodium-glucose cotransporter 2 inhibitors in alleviating neurocognitive impairment in diabetic mice
Source: Psychopharmacology (Berl). 2023 Mar 4;240(4):983–1000. doi: 10.1007/s00213-023-06341-7 (PMC10006050; doi:10.1007/s00213-023-06341-7)
Supplement: Supplementary file 1 — Supplementary file1 (DOCX 21 KB) [file 213_2023_6341_MOESM1_ESM.docx]

| **Empagliflozin Dapagliflozin** |
| --- |
| **Improvement of the short-term** Yes No  **memory deficits** |
| **Improvement of the long-term** No Yes  **memory deficits** |
| **Increased level of BDNF** Yes Yes |
| **Increased level of NT4** Yes No |
| **Increased expression of *Bdnf*** Yes Yes |
| **Increased expression of *App*** Yes (only in PFC) Yes |
| **Increased expression of *Snca*** Yes (only in PFC) Yes (only in HPC) |

PFC – the prefrontal cortex

HPC - hippocampus
